# S5. EFFECTS OF EARLY LIFE ADVERSITY ON IMMUNE FUNCTION AND COGNITIVE PERFORMANCE IN YOUTHS WITH AND WITHOUT EXPERIENCE OF PSYCHOTIC SYMPTOMS

**DOI:** 10.1093/schbul/sby018.792

**Published:** 2018-04-01

**Authors:** Jessica Holland, Maria Dauvermann, Derek Morris, Stan Zammit, Golam Khandaker, Gary Donohoe

**Affiliations:** 1 National University of Ireland, Galway; 2 University of Bristol; 3 Cambridge University

## Abstract

**Background:**

Early life adversity (ELA), including physical abuse or neglect and emotional abuse or neglect, is a significant risk factor for schizophrenia. Changes in cognitive function, and in particular social cognition, are also associated with this disorder. In psychosis, ELA and cognitive deficits have, separately, been associated with an increased immune response. In this study we sought to determine whether ELA’s might affect cognitive performance and if so, whether these affects were mediated via an impact on immune response.

**Methods:**

We investigated the relationship between ELA, immune response and cognition in the Avon Longitudinal study of parents and children (ALSPAC; n~5,000). ELA was defined in terms of the experience of physical abuse or neglect, emotional abuse or neglect, witnessing domestic violence, and harsh parenting before the age of 5 years. Social cognition was defined in terms of performance on theory of mind while general cognitive ability was defined in terms of IQ. Immune function was measured using C-reactive protein and Interleukin-6. Analysis was run both for the full sample and for individuals presenting with a history of psychotic symptoms at age 12.

**Results:**

Early life adversity was associated with poorer performance on a range of both general and social cognitive measures. Increased immune activation was associated with cognitive performance, but was not observed to mediate the effects of ELA on cognition. Comparable findings were observed in children presenting with and without psychotic symptoms.

**Discussion:**

While increased immune response has been associated with both early life adversity and cognitive impairment, this response was not observed to mediate the relationship between these two variables. Alternative hypothesis for the mechanism by which ELA may result in poorer cognitive performance, including attachment related effects, will be discussed.

